# Synchronous Surgical Treatment of Lower Eyelid Involutional Entropion and Ptosis

**DOI:** 10.1155/2018/2478646

**Published:** 2018-11-11

**Authors:** D. Vasakos, E. Nakos, C. Sioulis

**Affiliations:** 424 General Military Training Hospital of Thessaloniki, Thessaloniki, Greece

## Abstract

**Background:**

Involutional entropion and upper eyelid ptosis are common eyelid diseases in the elderly population. They represent a frequent cause of discomfort and often result in significant visual and functional impairment. The surgical management of these disorders includes various treatment options and techniques and is usually carried out in multiple time sessions.

**Case Report:**

We report the case of a 72 year old female patient, suffering from right eye involutional lower eyelid entropion and ptosis, who was treated synchronously for both conditions, by applying the lateral tarsal strip procedure and the levator resection technique.

**Conclusion:**

The synchronous treatment of involutional entropion and ptosis is an alternative treatment strategy, which could potentially improve surgical outcome, while reducing postoperative recovery time and treatment costs.

## 1. Introduction

Eyelid ptosis and involutional entropion are frequent eyelid disorders affecting people of advanced age. The prevalence of involutional entropion is about 2% in the elderly population [1, 2]. It is more frequent in whites and in females, due to anatomical differences compared to males, and its incident increases with age [2–6].

To date no sufficient data exist regarding the incidence of upper eyelid ptosis [7, 8]. Limited studies suggest that it may be around 2% in people over 60 years of age [9]. The cause of ptosis may be aponeurotic (most common form), traumatic, congenital, mechanical, neurogenic, and myogenic [10].

Involutional entropion may cause significant discomfort to the patient. Pathological findings may include lateral and medial canthal tendon laxity, lower retractor laxity, dry eye syndrome, chronic blepharitis, and chronic conjunctivitis [2].

Ptosis may cause a variety of symptoms such as blurred vision, tearing/epiphora, or an impaired upper visual hemifield, which often results in an abnormal head posture of the patient [1]. Typical clinical findings in aponeurotic ptosis may include good levator muscle function, deep upper eyelid sulcus, and upper eyelid dermatochalasis [11]. Blepharoptosis usually deteriorates by the end of the day, as a result of overexhaustion of Muller's muscle [1, 11, 12].

The laxity of the lateral and medial canthal tendon and false insertion of the lower retractors are the main causative factors of senile entropion [2, 12]. Other involutional changes such as atrophy of the orbital fat and the relaxation of the ligamentous support may also be important contributing factors [1, 4].

The main pathogenesis mechanism of blepharoptosis is involutional changes at the levator aponeurosis [10]. The levator muscle becomes thin and weak, which results in functional impairment and ptosis [1, 7]. Disinsertion or dehiscence of the levator aponeurosis due to chronic inflammatory conditions and intraocular surgery (eyelid speculum trauma) represents another important cause [1, 7].

Surgical techniques for the treatment of involutional entropion include the lateral tarsal strip procedure [13], the use of transverse everting sutures [1, 14], retractor plication [1, 15], transverse lid split [1], and the Wies procedure [14, 16].

Ptosis is usually treated by applying the levator resection technique [1], the Fasanella-Servat procedure [17], the Muller muscle–conjunctival resection procedure [18], or the frontalis slings operation, if there is poor or absent levator function [19].

We describe the case of a woman suffering from right eye involutional lower eyelid entropion and right upper eyelid ptosis, who was successfully treated synchronously for both conditions, by applying the lateral tarsal strip procedure and the levator resection technique.

## 2. Case Report

A 72 year old Caucasian female was admitted to our clinic with unilateral chronic symptoms of blurred vision, increased tearing, and foreign body sensation. The clinical examination revealed periocular dermatochalasis, lower eyelid laxity, dry eye disease, chronic blepharitis, chronic conjunctivitis, and superficial punctate keratopathy. A diagnosis of right eye upper eyelid ptosis and lower eyelid involutional entropion was made. A decision for the synchronous surgical treatment of both conditions followed, by using the lateral tarsal strip procedure and the levator resection technique. During the preoperative assessment the levator muscle's function and the severity of the ptosis were evaluated. The levator function was good and the severity of the ptosis was moderate (3mm). In addition, the clinical evaluation of the lower eyelid showed no punctum horizontal displacement.

The surgical procedure for both conditions was carried out under local anesthesia. The lower eyelid entropion was treated first. A lateral canthotomy and transection of the lateral canthal tendon were performed. The eyelid was then divided into anterior and posterior lamellae. A tarsal strip was fashioned from the posterior lamella and was then sutured to the periosteum at the lateral orbital wall, by using 5-0 ethibond double spatula sutures. Wound closure was achieved, by using absorbable 6-0 sutures (Vicryl), first for the orbicularis muscle and finally for the skin tissue.

Then the levator resection technique was performed in order to correct the ptosis. An incision, through the skin and orbicularis muscle, along the eyelid crease, was made. Dissection through the orbital septum followed. A double armed 5-0 ethibond suture was then placed through the anterior surface of the upper tarsus. Each of the needles was then placed through the healthy/homogenous part of the levator aponeurosis. The procedure led to the augmentation of the levator function.

The patient received topical antibiotic and corticosteroid treatment postoperatively for 10 consecutive days and was clinically assessed thereafter for the following 2 years.

The procedure performed resulted in the restoration of the upper and lower eyelid normal anatomy as well as in a significant reduction of the patient's discomfort and symptoms. During the 1-year and 2-year follow-up clinical assessments no clinical signs of recurrency were found. The pre- and postoperative (at 1 year follow up) VFQ-25 questionnaire revealed significant improvement of the patient's quality of life (questionnaire developed by RAND and funded by NEI and translated and validated in the Greek language according to the instructions by RAND).

## 3. Discussion

Senile entropion and aponeurotic ptosis are common eyelid disorders in the elderly population. They represent a frequent cause of significant eye discomfort. Both diseases are caused by complex involutional processes. Their surgical management, when applying the lateral tarsal strip and the levator resection techniques, is generally simple and in most cases produces good and long-lasting results.

The surgical procedures involved are usually performed separately and can be a significant burden for the patient, as postoperative recovery time and discomfort are often important disability factors. The adherence of the patient to potential future therapies may also be lower, if the surgical procedures involved are performed asynchronously. Moreover, if the life expectancy in the developed world increases, more people are expected to suffer from such diseases in the future. As a result, the treatment costs will be higher for the healthcare systems. Therefore it is important to find ways in which the suffering of the patient is less and the treatment becomes more cost-efficient.

In our case, the successful surgical management of the involutional entropion and the aponeurotic ptosis led to a significant reduction of the patient's postoperative discomfort and total recovery time, as both diseases were treated synchronously. The VFQ-25 questionnaire also showed significant improvement of the patient's quality of life. In addition, the synchronous procedure proved to be cost-efficient, due to the shorter time needed for the surgery completion and for the patient's hospitalization.

The synchronous surgical treatment of involutional entropion and aponeurotic ptosis is an alternative way to treat these frequent eyelid disorders. The lateral tarsal strip and the levator resection techniques are surgical procedures that can be easily performed by the eye surgeon. The synchronous surgical treatment might improve the patient's adherence to therapy and could be more cost effective for the healthcare system, while causing less postoperative disability.

## Supplementary Materials

Supplementary Materials (1) Preoperative clinical picture of the patient showing moderate upper eyelid ptosis and lower eyelid entropion. (2) Clinical picture of the patient 2 years postoperatively demonstrating successful and enduring anatomical and functional restoration of the upper and lower eyelids.
