# Supplementary figures and images for: Synchronous Surgical Treatment of Lower Eyelid Involutional Entropion and Ptosis
Source: Case Rep Ophthalmol Med. 2018 Nov 11;2018:2478646. doi: 10.1155/2018/2478646 (PMC6252241; doi:10.1155/2018/2478646)

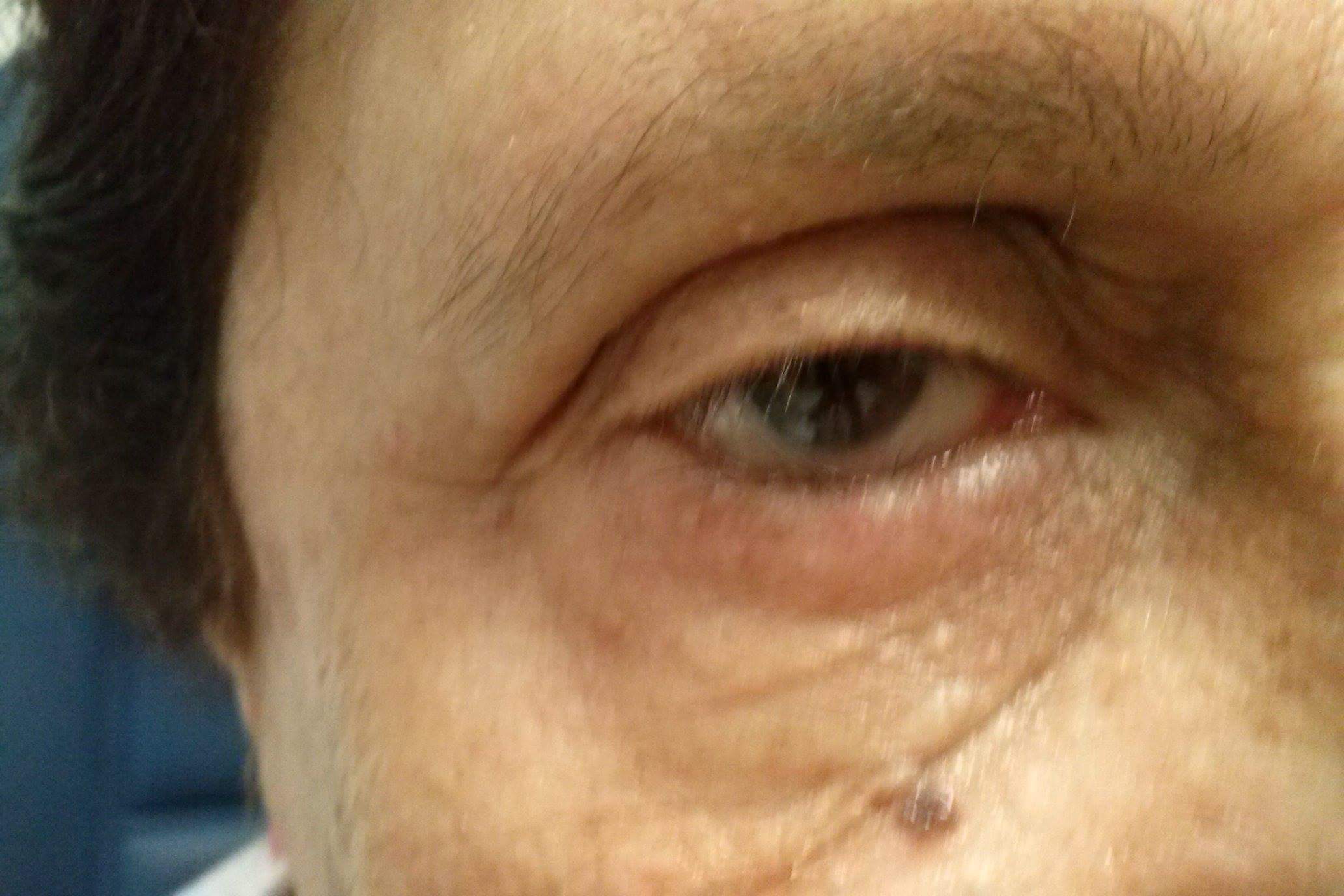

Supplement: Supplementary Materials — (1) Preoperative clinical picture of the patient showing moderate upper eyelid ptosis and lower eyelid entropion. (2) Clinical picture of the patient 2 years postoperatively demonstrating successful and enduring anatomical and functional restoration of the upper and lower eyelids. [file 2478646.f1.zip › 1.pre-operative clinical picture of the patient.jpeg]

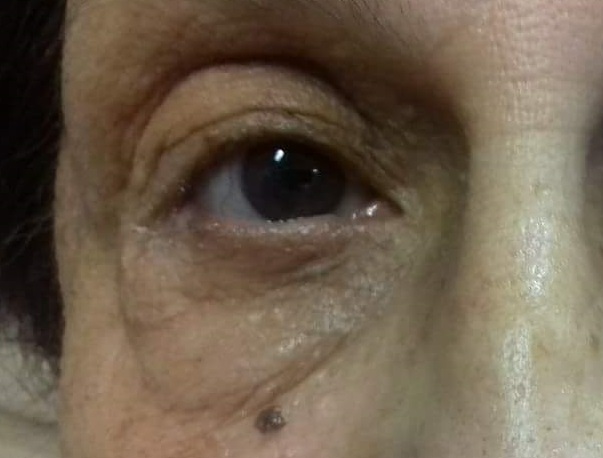

Supplement: Supplementary Materials — (1) Preoperative clinical picture of the patient showing moderate upper eyelid ptosis and lower eyelid entropion. (2) Clinical picture of the patient 2 years postoperatively demonstrating successful and enduring anatomical and functional restoration of the upper and lower eyelids. [file 2478646.f1.zip › 2.clinical picture of the patient after 2 years.jpeg]
